# Supplementary material for: Impact of iSupport on improving knowledge on dementia and dementia care among family caregivers to persons with dementia and formal caregivers in Bangladesh: Protocol for a non-randomized feasibility study
Source: PLoS One. 2025 Jun 13;20(6):e0326207. doi: 10.1371/journal.pone.0326207 (PMC12165409; doi:10.1371/journal.pone.0326207)
Supplement: Attachment — Submitted filename: Response to Reviewers_250319.docx [file pone.0326207.s003.docx]

## **Response to Reviewer #1**

*The manuscript effectively highlights the growing burden of dementia in LMICs like Bangladesh and the lack of adequate caregiving support. The introduction clearly establishes the need for scalable interventions such as the WHO iSupport manual. The research questions are well-defined, focusing on improving dementia knowledge and caregiving outcomes, and they are justified within the context of the limited literature on dementia care in Bangladesh.*

**Comment 1*:*** *However, while iSupport was originally designed for informal caregivers, the inclusion of formal caregivers in the study is not sufficiently justified, particularly since the content remains unchanged. Formal caregivers might find the examples used in iSupport irrelevant, potentially leading to high attrition or non-adherence, especially given that the eligibility criteria do not require them to be dementia caregivers. Adaptations specific to formal caregivers may be necessary, and as the project is already in progress, the authors should discuss how they plan to address this issue.*

**Response 1:** Thank you for your valuable comment. We acknowledge that the content in the iSupport manual may be less relevant for formal caregivers, as the manual was originally designed for family caregivers of persons with dementia (PWD). However, given the limited availability of dementia-specific caregiver training in Bangladesh and of trained caregivers, we believe that iSupport can serve as a valuable introductory resource to improve caregivers' knowledge and understanding of dementia care. Additionally, their employer noted that some formal caregivers already have experience working with PWD. Training can further enhance their ability to recognize dementia symptoms in the people they care for and manage them more effectively. Given the fact that most of their clients are older persons, and the higher prevalence of dementia among older adults in Bangladesh, testing iSupport’s feasibility among formal caregivers can be beneficial.

To maintain engagement and mitigate the risk of non-adherence, a weekly text message is sent on WhatsApp as a gentle reminder to formal caregivers. This is now mentioned in the section describing the **iSupport intervention** (**lines 226-227, page 12** of the **revised manuscript with track changes**) under **Materials and methods**.

**Comment 2:** *Study Design and Methodology*
*The study protocol outlines a robust methodology that combines quantitative and qualitative approaches. The use of validated tools such as DKAS, MCSI, and GAD-7 enhances credibility. However, several methodological concerns should be addressed:* **Comment 2.1.** *The non-randomized sampling method and reliance on purposive sampling may introduce selection bias, potentially limiting generalizability. While the authors acknowledge these as limitations, their impact on the validity of the findings should be discussed more explicitly.*

**Response 2.1:** Thank you for your insightful comment. We acknowledge that the non-randomized design and purposive sampling method may introduce selection bias, which could limit the generalizability of our findings. This has now been mentioned in the **Limitations** sub-section (**lines 482-483, page 24** of the **revised manuscript with track changes**) in the **Discussion** section.

**Comment 2.2.** *The recruitment of formal caregivers from a single agency may not capture the diversity of caregiving experiences in Bangladesh. Expanding recruitment to multiple agencies or organizations would improve the representativeness of the sample.*

**Response 2.2:** We agree with the reviewer’s observation. However, expanding recruitment to multiple agencies may not be feasible due to financial and logistical constraints. Moreover, the iSupport intervention involves an extensive reading material and use of an online platform, requiring participants to possess a certain level of education and digital literacy. To our knowledge, there are 26 fully operational caregiving service providers in Bangladesh, with only three providing similar level of service in Bangladesh.

**Comment 2.3.** *The eligibility criterion for formal caregivers—“experienced in providing care to older persons”—is vague. Defining a minimum number of years of caregiving experience would improve clarity and ensure consistency in participant selection.*

**Response 2.3:** Thank you for your valuable comment. We have also reconsidered this criterion and removed it. This change is reflected in the **Eligibility** sub-section of **Participant selection and recruitment** (**line 190, page 10** of the **revised manuscript with track changes**) under **Materials and methods**.

**Comment 2.4.** *One of the research questions asks whether the impact of iSupport varies with the level of caregiving skills among formal caregivers. However, the manuscript does not specify how caregiving skill levels will be assessed. A clear operational definition or assessment framework should be provided.*

**Response 2.4:** The agency from which formal caregivers were recruited classifies their caregivers into three levels: Level 1 (basic-care), Level 2 (general-care), and Level 3 (advanced-care). This classification is based on their practical skills, work ethics, and educational qualifications, with a minimum requirement of a Secondary School Certificate (10 years of schooling). The categorization of formal caregivers are indicators of their skills. This is now indicated in the **Sample size** section under **Materials and methods** (**lines 359-363, page 18** of the **revised manuscript with track changes**).

**Comment 3:** *Adaptation of iSupport to Bangladesh*
*The authors describe the translation and adaptation of iSupport for the Bangladeshi context, including stakeholder consultations and pre-testing. However, the WHO has developed an Adaptation and Implementation Guide (World Health Organization, 2019), which provides structured recommendations for localization. The authors could consider aligning their adaptation process with this guide to ensure cultural and contextual relevance.*

**Response 3:** The adaptation of the Bangla iSupport manual followed most of the standard adaptation guidelines by WHO and international adaptation practices. The process began in November 2023 and concluded in August 2024. The adaptation process involved: (1) content translation, (2) linguistic and cultural adaptation, (3) stakeholder engagement and approval, and (4) fidelity assessment. The cultural adaptation process has now been detailed in the **Adaptation** sub-section of the **iSupport intervention** section under **Materials and methods** (**lines 238-277, pages 12-14** of the **revised manuscript with track changes**).

**Comment 4:** *Intervention and Digital Accessibility
More details are needed regarding how iSupport will be presented online. For example:*

- *Will it be structured as an e-book, a blog, or an interactive learning platform?*
- *How will users navigate different modules and lessons?*
- *Are there any interactive elements, quizzes, or progress-tracking features?*

**Response 4:** The iSupport manual is hosted on a dedicated website, where participants must log in using credentials provided by the research team. The platform allows participants to access and navigate modules in any order, add and save personal notes on each page, and complete quizzes at the end of each section. Although this platform does not provide real-time quiz feedback, like the original manual, the answers are available at the end of each section. The saved notes, along with the participants’ progress can be reviewed by the research team. Details on how iSupport will be presented online has now been added in the second paragraph of the **iSupport intervention** section in **Materials and methods** (**lines 212-217, page 11** of the **revised manuscript with track changes**).

**Comment 5:** *Additionally, digital literacy and internet access barriers could impact adherence. A brief discussion on how these challenges will be addressed—such as providing offline materials or digital literacy training—would strengthen the feasibility argument.*

**Response 5:** To address potential digital literacy and internet access barriers, caregivers will be asked during the interview about their internet access, the type of devices they own, and their ability to use the internet independently. Additionally, formal caregivers will receive a brief training session after their baseline survey where a member of the research team will demonstrate how to use the website (mentioned in **lines 194-198** on **pages 10-11**, and **lines 224-226** on **page 12** of the **revised manuscript with track changes**). During the training, formal caregivers will be asked to log in to the website themselves and the trainer will observe their ability to navigate the website. If needed, they are given a paper-based version of the manual (mentioned in **line 199-200, page 11** of the **revised manuscript with track changes**). This process of assessing and addressing challenges of digital literacy is detailed in the **Eligibility** section under **Participant selection and recruitment** within **Materials and methods** (**lines 194-200 and 224-226** on **pages 10-12** of the **revised manuscript with track changes**).

For family caregivers, digital literacy and internet access are prerequisites for participation. Our eligibility criteria specify that participants must be able to browse the internet on computers and/or handheld devices and have access to the internet at their own cost. These criteria are outlined in under the **Eligibility** section in **Participant selection and recruitment** within **Materials and methods** (**lines 185-186, page 10** of the **revised manuscript with track changes**).

**Comment 6:** *Statistical Analysis and Data Management*
*The statistical analysis plan is generally sound, but further details are needed on:*
**Comment 6.1.** *How missing data will be handled in the analysis.*

**Response 6.1:** Thank you for your comment. Since our survey questionnaire does not include any culturally or personally sensitive questions, we anticipate minimal missing data. Missing values will be handled using simple imputation techniques. For continuous variables, missing values will be replaced with the mean, while for categorical variables, the modal category will be used. This is now mentioned in the **Data analysis plan** in **Materials and methods** (**lines 424-425** on **page 21** of the **revised manuscript with track changes**).

**Comment 6.2.** *The justification for the high attrition rate assumption—providing relevant citations for similar studies would strengthen this estimate.*

**Response 6.2:** Thank you for your comment. We have cited a study protocol to test the impact of the iSupport manual in Australia who also assumed a 40% attrition rate. The citation has been added in the **Sample size** section under **Materials and methods** (**line 354, page 18** of the **revised manuscript with track changes**).

**Comment 7:** *The manuscript also describes strong data security measures, including the use of SurveyCTO and password-protected storage. However, it does not explicitly state whether the data will be made publicly available post-study. Clarifying the data sharing plan would improve research transparency.*

**Response 7:** We appreciate your suggestion regarding data sharing. De-identified participant data will be made publicly available in accordance with ethical guidelines and data protection regulations. The data will be uploaded as supplementary information, with appropriate measures to ensure participant confidentiality. This has now been mentioned in the **Data management** section under **Data collection plan** within **Materials and methods** (**lines 410-412, page 20** of the **revised manuscript with track changes**).

**Comment 8:** *Qualitative Analysis
While the qualitative methods are well-detailed, the manuscript does not specify strategies for ensuring reliability in coding and analysis. Providing details on how themes will be identified, how inter-coder reliability will be maintained, and whether software will be used for analysis would enhance methodological rigor.*

**Response 8:** Thank you for the comment. No software will be used for data analysis. We will analyze data using inductive content analysis. The process of inductive content analysis includes open coding, creating subcategories, and abstraction to categories. Findings will be discussed back and forth among the authors until they reach a consensus, to increase validity. Transferability will be ensured through a detailed description of the data collection process and a sensitivity to the context in which data will be collected. This has now been detailed in the last paragraph of the **Data analysis plan** under **Materials and methods** (**lines 430-434, page 21** of the **revised manuscript with track changes**).

**Comment 9:** *Discussion and Broader Implications
The discussion section primarily focuses on justifying the methodology and acknowledging potential challenges. However, it could be strengthened by:*
**Comment 9.1.** *Expanding on the broader implications of the findings for dementia care policy and practice in Bangladesh.*

**Response 9.1:** Currently there is a lack of policy on dementia care and guidelines on practice in Bangladesh. Findings of this pioneering study will help structure competence building among family and formal caregivers in their role for caring for PWD. This is one of the few studies or maybe even the first one in Bangladesh which will help to create a knowledge base on how to care for a PWD living at home and even self-care strategies for the caregiver. This text has been added as the second paragraph of the **Discussion** section (**lines 450-454, page 22** of the **revised manuscript with track changes**).

**Comment 9.2.** *Addressing how the study results could inform future adaptations of iSupport or similar interventions in other LMICs.*

**Response 9.2:** Thank you for your comment. This feasibility study will provide insights into the feasibility of implementing the iSupport manual in Bangladesh, where caregiving is largely informal, with most PWD receiving care from a family member with little knowledge or paid individual with limited educational qualifications. The findings could inform future adaptations of iSupport or similar interventions, including the development of a tailored version of iSupport for formal caregivers. Lessons learned from the adaptation process, combined with feedback from stakeholders and participants, will help refine the content and delivery modalities (i.e., online, paper-based, or in-person training) to increase cultural relevance, ease of understanding and accessibility in resource-constrained settings. These insights will help lay the groundwork for broader trials and scale-up efforts in Bangladesh and other LMICs. This text has been added as the last paragraph of the **Discussion** section (**lines 470-478, page 23** of the **revised manuscript with track changes**).

**Comment 10:** *Ethical and Financial Disclosure
The financial disclosure statement appears contradictory to the acknowledgment section. This should be clarified to ensure consistency.* **Response 10:** Thank you for pointing this out. The authors received no external funding for this research project. The research was funded internally by the SAJIDA Foundation. The **Acknowledgement** section has now been revised to ensure consistency with the financial disclosure statement (see **lines 491-492** on **page 24** of the **revised manuscript with track changes**).

*Overall, this is a well-constructed study protocol that addresses a significant gap in dementia care research in Bangladesh. The study's potential impact is notable, but addressing the outlined concerns—particularly regarding formal caregiver inclusion, intervention delivery, data transparency, and methodological details—will further enhance its rigor and relevance.*

## **Response to Reviewer #2**

**Comment 1:** *This study aims to assess the feasibility of using the WHO iSupport (printed) manual with family and formal carers in Bangladesh. There is no mention how the manual would be used in an "online" program as mentioned in the paper.*

**Response 1:** Thank you for your valuable comment. The iSupport manual is hosted on a dedicated website, where participants must log in using credentials provided by the research team. The platform allows participants to access and navigate modules in any order, add and save personal notes on each page, and complete quizzes at the end of each section. Although this platform does not provide real-time quiz feedback, like the original manual, the answers are available at the end of each section. The saved notes can be reviewed by the research team. Additionally, the research team can track participants' progress, and access saved notes through website analytics. Details on how iSupport will be presented online has now been added in the second paragraph of the **iSupport intervention** section in **Materials and methods** (**lines 212-217, page 11** of the **revised manuscript with track changes**).

**Comment 2.1.** *It is also not clear how the manual would be translated into the local language and culturally adapted.*

**Response 2.1:** We appreciate your comment. The adaptation of the Bangla iSupport manual followed the standard adaptation guidelines made available by WHO and international adaptation practices. The process began in November 2023 and concluded in August 2024. The adaptation process involved: (1) content translation, (2) linguistic and cultural adaptation, (3) stakeholder engagement and approval, and (4) fidelity assessment. The cultural adaptation process has now been detailed in the second and third paragraphs under the **Adaption** sub-section of the **iSupport intervention** section under **Materials and methods** (**lines 238-277, pages 12-14** of the **revised manuscript with track changes**).

**Comment 2.2.** *Has permission from the WHO been sought for this adaptation?*

**Response 2.2:** According to WHO’s directives, no formal licensing is required. The original iSupport manual by WHO mentions:

“Under the terms of this licence, you may copy, redistribute and adapt the work for non-commercial purposes, provided the work is appropriately cited, as indicated below. In any use of this work, there should be no suggestion that WHO endorses any specific organization, products or services. The use of the WHO logo is not permitted. If you adapt the work, then you must license your work under the same or equivalent Creative Commons license. If you create a translation of this work, you should add the following disclaimer along with the suggested citation: “This translation was not created by the World Health Organization (WHO). WHO is not responsible for the content or accuracy of this translation. The original English edition shall be the binding and authentic edition.”

Despite this, the iSupport team at WHO was informed about the adaptation of the iSupport manual for the specific context of Bangladesh. The iSupport team shared the adaptation guideline with us. This has now been mentioned in the **Adaptation** sub-section of the **iSupport intervention** section under **Materials and methods** (**lines 240-242, pages 12-13**). Furthermore, most of the authors of this protocol are members of the WHO iSupport network. Adaptation guidelines by WHO were followed, which is detailed in the **Adaptation** sub-section of the **iSupport intervention** section under **Materials and methods** (**lines 238-277, pages 12-14** of the **revised manuscript with track changes**).

**2.3.** *Possibly the cultural adaptation needs to be tested in a pilot study in a smaller sample of carers and professionals, to see if is understandable and useable, using qualitative methods.*

**Response 2.3:** Thank you for your comment. The Bangla adaptation was reviewed by experts in the field and individuals proficient in both Bangla and English, and tested in a stakeholder meeting with formal caregivers. This has now been mentioned detailed in the third paragraph under the **Adaptation** sub-section of the **iSupport intervention** section under **Materials and methods** (**lines 260-267, pages 13-14** of the **revised manuscript with track changes**).

**Comment 3.1.** *The primary aim of the study is to see if iSupport improves the carers knowledge about dementia. While it is clear that the family carers are managing PWD, it is unclear who the formal carers are, and if they are actually managing PWD.*

**Response 3.1:** Formal caregivers recruited for this study are caregivers who are employees of an agency providing home-care services. It has been mentioned in the **Eligibility** section in **Participant selection and recruitment** under **Materials and methods** (**line 187, page 10** of the **revised manuscript with track changes**) that formal caregivers do not have to be caring for PWD. However, during the survey, formal caregivers will be asked if they have provided care or are currently providing care to a PWD.

**Comment 3.2.** *It is also not clear why the authors are recruiting a larger sample of formal carers and why they are looking for male vs female differences in the outcome. The two samples could be equal, based on the sample size calculation?*

**Response 3.2:** The minimum required sample size for each group was estimated to be 44. We plan to recruit a total of 132 formal caregivers to represent 44 formal caregivers in each caregiver category (i.e., Level 1, Level 2, and Level 3). This is mentioned in the **Sample size** section under **Materials and methods** (**lines 359-365** on **page 18** of the **revised manuscript with track changes**).

This larger sample size will enable us to test for potential differences in dementia knowledge between male and female formal caregivers before and after the intervention, as well as to explore whether baseline knowledge differs between the two groups. This will allow us to detect statistically significant differences in outcomes. This has now been added in the **Sample size** section under **Materials and methods** (**lines 365-367** on **page 18** of the **revised manuscript with track changes**).

**Comment 4:** *There is no mention about the translation and validation of all the study instruments into the local language. No references are provided if this has already been done before the study.*

**Response 4:** Thank you for your feedback. The GAD-7 and CESD-10 tools used in this study have been validated in Bangla. All other study instruments mentioned were translated into Bangla by the research team. The translation process included pre-testing the questionnaire with caregivers, and making adjustments based on their feedback. The **Study instruments** section in **Materials and methods** has now been revised to include the above-mentioned information and appropriate references for the validated GAD-7 and CESD-10 tools in Bangla (**lines 308-311, pages 15-16** of the **revised manuscript with track changes**).

**Comment 5:** *It is not clear why the iSupport manual would be available to the carers only for two months. What is the reason for this restriction, if at all, and how would the researchers ensure that the manual is not available after this two month period?*

**Response 5:** We decided on a two-month intervention period keeping with a time frame as per discussion with researchers in the UK who worked on adapting the iSupport manual for Bangla speakers in the UK. Additionally, we tested the manual and confirmed that the Bangladeshi adaptation of the manual can be read within two months.

Caregivers receive unique IDs and passwords to access the website after their baseline interview. At the end of the two-month period, the research team disables access, ensuring that the manual can no longer be accessed online. For formal caregivers who receive a printed version of the manual, they are required to return it during their end-line interview. The process of ensuring the manual is no longer accessible after the two-month intervention period is now briefly mentioned at the end of the last paragraph of the **iSupport intervention** section in **Materials and methods** (**lines 227-229, page 12** of the **revised manuscript with track changes**).

**Comment 6:** *Without a control group it may not be possible to study the feasibility or "effectiveness" of using the iSupport to test the secondary aims of improvement in caregiver skills, competency, distress, quality of life, etc.*

**Response 6:** We acknowledge that the absence of a control group limits our ability to assess iSupport’s effectiveness in achieving the secondary objectives. The inclusion of a control group was not feasible due to financial constraints and challenges in recruiting caregivers in Bangladesh. Formal caregivers, particularly those working in home-based or institutional settings, often have demanding schedules, making recruitment difficult. Family caregivers, on the other hand, may lack the time or flexibility to participate in such studies. Moreover, there is no identified pool of family caregivers, making it difficult to select a control group. We hope that the findings from this study will help inform the design of future trials with more rigorous evaluation strategies.

This has now been mentioned as a limitation in the **Limitation** sub-section in the **Discussion** (**lines 483-485, page 24** of the **revised manuscript with track changes**)**.**

**Comment 7:** *In the consent form there is mention that the formal carers would be monetarily compensated while the family carers would receive no money or incentives. No mention or reason for this is given in the paper itself.*

**Response 7:** Formal caregivers will receive monetary compensation as they are required to travel to the survey location and, in some cases, take leave from work to participate. Family caregivers will be surveyed at their homes at a time convenient for them, eliminating travel or work-related disruptions. Therefore, monetary compensation is not provided to family caregivers.
