# Supplementary material for: Impact of iSupport on improving knowledge on dementia and dementia care among family caregivers to persons with dementia and formal caregivers in Bangladesh: Protocol for a non-randomized feasibility study
Source: PLoS One. 2025 Jun 13;20(6):e0326207. doi: 10.1371/journal.pone.0326207 (PMC12165409; doi:10.1371/journal.pone.0326207)
Supplement: Attachment — Submitted filename: Response to Reviewer_250519.docx [file pone.0326207.s004.docx]

## **Response to Reviewer #1**

**Comment 1*:*** *Access to Intervention Materials (pg. 12, lines 228-229)*

*The authors state that “access to, both, online and printed versions will be restricted by disabling online access and taking back the printed manual before their end-line survey.” While the intention is understandable, removing the printed version does not fully guarantee restricted access, as participants could easily photocopy or retain copies of the manual during the intervention period. Unlike the online platform, which is more easily controlled via login access, printed materials cannot be secured in the same way. This should be acknowledged as a limitation of the study in the manuscript.*

**Response 1:** Thank you for your valuable comment. We acknowledge the possibility of participants photocopying or retaining copies of the printed manual. We have now mentioned this as a limitation in the last sentence of the **Limitations** sub-section (**lines 465-467, page 23** of the **revised manuscript with track changes).**

**Comment 2:** *Clarification Needed-Comment 2.2*

*In response to comment 2.2, the authors state: “To our knowledge, there are 26 fully operational caregiving service providers in Bangladesh, with only three providing similar level of service in Bangladesh.” The meaning of “similar level of service” is unclear. It would be helpful to specify what is meant by this - does it refer to the quality of training, type of clientele, scope of services, or caregiver qualifications? Please revise this sentence for clarity.*

**Response 2:** “Similar level of service” refers to the quality of caregiver training, overall scope of services provided, and caregiver qualifications. The social enterprise through which the formal caregivers are recruited is the only ISO-certified caregiving agency in Bangladesh, indicating alignment with international standards in training and labour law compliance. Moreover, the enterprise has strict criteria for employment and progress to higher levels, such as minimum 10 years of schooling, minimum one year experience at the agency, Diploma in Nursing, and performance evaluations, which ensure higher standard of caregiver qualifications and scope of service. This has now been briefly mentioned in the **Recruitment and consent process** sub-section of **Participant selection & recruitment** (**lines 173-175, pages 9-10** of the **revised manuscript with track changes**) under **Materials and methods**.

**Comment 3:** *Definition of Formal Caregivers (Comment 2.3)*

*The authors’ response to the concern about vague eligibility criteria for formal caregivers is not satisfactory. Removing the criterion is not an adequate solution or justification. Instead, the authors should consider incorporating a clearer and stricter definition of “formal caregivers,” such as minimum years of experience, specific care responsibilities, or formal employment status in caregiving services.*

**Response 3:** In this study, formal caregivers are defined by their employment status – specifically, as individuals employed by the selected social enterprise. Their eligibility as formal caregivers are further defined by the enterprise’s criteria, which include minimum 10 years of schooling to become a caregiver, and at least one year of experience within the organization for advanced care responsibilities. This has now been briefly mentioned in the **Eligibility** sub-section of **Participant selection and recruitment** (**lines 189-192, page 10** of the **revised manuscript with track changes**) under **Materials and methods**.

**Comment 4:** *Sentence Clarity (pg. 12, lines 238–241)*

*The following sentence is clunky and difficult to follow:*

*“This process followed the standard adaptation guidelines provided by WHO (38) upon informing them about the adaptation of the iSupport manual for the specific context of Bangladesh, and international adaptation practices (33,34) to ensure the manual’s suitability for Bangladeshi caregivers.”*

*I suggest restructuring it for clarity. For example:*

*“The adaptation process followed the standard guidelines provided by WHO (38), and the research team informed WHO about the adaptation for the Bangladeshi context. International adaptation practices (33,34) were also followed to ensure the manual’s relevance and suitability for caregivers in Bangladesh.”*

**Response 4:** Thank you for your suggestion. Accordingly, **lines 244-248** on **page 13** have been restructured in the **Adaptation** sub-section of **The iSupport intervention** section of **Materials and methods**.

**Comment 5:** *Data Availability Statement*

*The authors mention that de-identified participant data will be made publicly available as supplementary information. However, it remains unclear where this data will be published- whether as supplementary files alongside a results publication, in a specific data repository, or on an institutional platform. Please clarify the planned location and format for data sharing to meet transparency and open science standards.*

**Response 5:** Thank you for your comment. Data will be made publicly available as supplementary files in CSV format, accompanying the publication(s) of results in journals. We have now clarified this in the **Data management** section of the **Data collection plan** under **Materials and methods (line 388, page 19 of the revised manuscript with track changes)**.
